# Optimising Exome Captures in Species With Large Genomes Using Species‐Specific Repetitive DNA Blocker

**DOI:** 10.1111/1755-0998.14053

**Published:** 2024-12-18

**Authors:** Robert Kesälahti, Timo A. Kumpula, Sandra Cervantes, Sonja T. Kujala, Tiina M. Mattila, Jaakko S. Tyrmi, Alina K. Niskanen, Pasi Rastas, Outi Savolainen, Tanja Pyhäjärvi

**Affiliations:** ^1^ Department of Ecology and Genetics University of Oulu Oulu Finland; ^2^ Department of Forest Sciences University of Helsinki Helsinki Finland; ^3^ Biocenter Oulu University of Oulu Oulu Finland; ^4^ Natural Resources Institute Finland (Luke) Oulu Finland; ^5^ Institute of Biotechnology University of Helsinki Helsinki Finland

**Keywords:** c0t‐1 DNA, conifer, exome capture, large genome, *Pinus sylvestris*, repetitive DNA

## Abstract

Large and highly repetitive genomes are common. However, research interests usually lie within the non‐repetitive parts of the genome, as they are more likely functional, and can be used to answer questions related to adaptation, selection and evolutionary history. Exome capture is a cost‐effective method for providing sequencing data from protein‐coding parts of the genes. C0t‐1 DNA blockers consist of repetitive DNA and are used in exome captures to prevent the hybridisation of repetitive DNA sequences to capture baits or bait‐bound genomic DNA. Universal blockers target repetitive regions shared by many species, while species‐specific c0t‐1 DNA is prepared from the DNA of the studied species, thus perfectly matching the repetitive DNA contents of the species. So far, the use of species‐specific c0t‐1 DNA has been limited to a few model species. Here, we evaluated the performance of blocker treatments in exome captures of *Pinus sylvestris* , a widely distributed conifer species with a large (> 20 Gbp) and highly repetitive genome. We compared treatment with a commercial universal blocker to treatments with species‐specific c0t‐1 (30,000 and 60,000 ng). Species‐specific c0t‐1 captured more unique exons than the initial set of targets leading to increased SNP discovery and reduced sequencing of tandem repeats compared to the universal blocker. Based on our results, we recommend optimising exome captures using at least 60,000 ng of species‐specific c0t‐1 DNA. It is relatively easy and fast to prepare and can also be used with existing bait set designs.

## 1 Introduction

Large and highly repetitive genomes are common among different types of organisms, such as amphibians, fish and plants. Repetitive DNA sequences can account for close to 80% of the genome in some species, such as maize (Flavell et al. 1974). However, research interests usually lie within the non‐repetitive parts of the genome, as they are more likely functional and thus can inform research on adaptation, breeding, function and evolutionary history. Non‐repetitive parts are also bioinformatically less challenging than repetitive parts. Obtaining sequencing data from regions of interest in a cost‐efficient manner is a highly important aspect when working with large genomes. Whole‐genome sequencing (WGS) is a great tool for generating large quantities of sequencing data across the genome. However, obtaining enough sequencing depth at specific sites of interest for reliable downstream analyses easily becomes expensive and limits the sample size. A lot of sequencing effort in WGS is essentially being ‘wasted’ on repetitive DNA sequences.

Targeted sequencing is a cost‐effective alternative to WGS. Restriction‐site associated DNA sequencing (RADSeq; Miller et al. 2007) and genotyping by sequencing (GBS; Elshire et al. 2011) use restriction enzymes to cut DNA at specific sites, followed by sequencing of adjacent regions. Restriction‐based methods do not necessarily require any prior knowledge of the studied species. A major downside is that they produce a lot of missing data among individuals due to, for example, mutated or missing restriction sites. RNA sequencing can also be utilised in targeted genome analyses, as it limits sequencing to the exons of expressed genes. Further, RNA sequencing data can be used to construct a transcriptome which can be utilised for other targeted sequencing methods, such as exome capture and amplicon sequencing.

Exome capture (Ng et al. 2009) uses biotinylated baits, designed based on a transcriptome or a reference genome, to capture and sequence a set of pre‐selected exons. Usually, the whole exome can be targeted, as the coding regions only constitute a few percent of the total genomic DNA. Exome captures produce fewer missing data between individuals compared to RNA‐sequencing and restriction‐based methods. However, it requires previous knowledge of the studied species to produce a reliable set of baits. Amplicon sequencing is an alternative method for sequencing specific genomic regions. It utilises baits with polymerase chain reaction (PCR) to produce ultra‐deep sequencing of the targeted regions that are usually smaller than in exome captures. Amplicon sequencing requires less starting DNA material, has simpler DNA preparation steps and produces higher on‐target rates, but lower uniformity, compared to exome captures (Samorodnitsky et al. 2015). Even more specific methods, such as primer extension target enrichment (PETE) and duplex sequencing (Kennedy et al. 2014; Schmitt et al. 2012), are available when working with a smaller set of targets. These methods achieve lower error rates than amplicon sequencing and exome capture but are currently too expensive to use on a larger scale.

When using target enrichment methodologies, off‐target repetitive DNA can end up being sequenced for two reasons. First, different types of short tandem repeats (microsatellites, minisatellites and satellites) can non‐specifically bind to the baits blocking the binding to the exons of interest. Second, the baits are usually much shorter than the genomic DNA fragments, and a large proportion of the fragment remains single‐stranded even after binding of the bait. Consequently, another genomic DNA fragment containing repetitive DNA sequences can bind to it, leading to the sequencing of the whole complex.

Exome captures use c0t‐1 DNA to block repetitive DNA sequences from binding to capture baits and bait‐bound genomic DNA fragments during the hybridisation reaction. C0t‐1 DNA contains a large pool of different types of repetitive DNA sequences of various lengths. It has originally been used to study DNA reassociation kinetics to determine the repetitive DNA contents of genomes within various species, such as maize, pea and sunflower (Flavell et al. 1974). C0 stands for the initial concentration of DNA and t for the DNA reassociation time in seconds (Britten, Graham, and Neufeld 1974). The amount of reassociation is measured relative to the original DNA concentration. Repetitive DNA reassociates faster than unique DNA sequences due to the higher probability of finding complementary sequences. Thus, repetitive DNA was labelled as low c0t and unique DNA as high c0t. Nowadays (low) c0t‐1 DNA is used in the hybridisation reaction of exome capture to reduce non‐specific binding, as c0t‐1 DNA binds to the repetitive DNA sequences of the genomic DNA. Species‐specific c0t‐1 DNA is routinely used in the exome captures with model species such as humans and mice. Researchers working with non‐model species only have different types of universal blockers available. These universal blockers consist of various highly conserved repetitive DNA sequences and thus do not perfectly reflect the repetitive DNA landscape found within the studied species. The use of universal blockers can potentially lead to increased quantities of non‐specific binding in exome captures. Large amounts of non‐specific binding lead to a decreased number of unique reads originating from the target regions. Producing species‐specific c0t‐1 DNA is a relatively easy and inexpensive alternative to using universal blockers and has been used with good results in amphibians with large genomes (McCartney‐Melstad, Mount, and Shaffer 2016). Despite its potential, species‐specific c0t‐1 DNA is still not used widely in exome captures of non‐model species.

In this study, we evaluated the performance of three different treatments for blocking the binding of repetitive DNA sequences to the capture baits and capture‐bound genomic DNA fragments in exome captures of *Pinus sylvestris*, a widely distributed conifer species (Durrant, De Rigo, and Caudullo 2016) with a large (> 20 Gbp) and highly repetitive genome. One of the treatments used a commercial universal blocker and two of the treatments used species‐specific c0t‐1 DNA, prepared using an optimised protocol for *P. sylvestris* , in two different quantities: 30,000 and 60,000 ng. We developed a novel bait set for *P. sylvestris* based on earlier transcriptome assembly (Ojeda et al. 2019), and used this set to evaluate the performance of the different treatments. We used only a single genotype in all exome captures to avoid the effects of genetic variation between different individuals. Whole‐genome sequencing was conducted to estimate how much and what type of repetitive DNA is being sequenced with no blocking treatment and capture. Our main research questions were: (I) Does the use of species‐specific c0t‐1 DNA improve exome captures by producing more unique reads compared to commercial blockers and (II) does doubling the recommended amount of species‐specific c0t‐1 DNA produce more unique reads and reduce the capture of repetitive DNA sequences?

As conifer genomes are large and repetitive, they have been lacking in genomic resources in comparison to many other plants and, for example, angiosperm forest trees. Whereas the *Arabidopsis thaliana* (2000) and Poplar genomes (Tuskan et al. 2006) were published in early 2000s, the first gymnosperm complete genome sequence, that of *Picea abies* was published only in 2013 (Nystedt et al. 2013), followed by, for example *Pinus taeda* (Neale et al. 2014; Wegrzyn et al. 2014) and *Pinus lambertiana* (Stevens et al. 2016). Note that the genome of the Chinese Pine ( *Pinus tabuliformis* , Niu et al. 2022) was the first chromosome‐level pine assembly, and was published after the bait design stage of this study.

## 2 Materials and Methods

### 2.1 Bait Set Design

Exome capture baits were designed based on *P. sylvestris* gene‐level SuperTranscript assembly (https://www.ncbi.nlm.nih.gov/nuccore/GILP00000000.1; Ojeda et al. 2019). Mosaic SuperTranscripts, multi‐copy genes, transcripts originating from organelle genomes and transcripts containing heterozygous SNPs in haploid megagametophyte samples were excluded from the list of transcripts, as described by Ojeda et al. (2019). Transcripts were also filtered based on the expression levels, requiring the sum of TPM (Transcripts Per Million) counts across all 30 RNA‐seq samples (Ojeda et al. 2019) to be above ten (https://figshare.com/articles/dataset/Pinus_sylvestris_assembly_Trinity_guided_gene_level_information/13109492/1). The remaining 108,861 transcripts were considered potential targets for baits. The list of potential targets includes a hand‐curated list of 2435 candidate genes for phenology and for primary and secondary metabolism pathways active during heartwood formation (File S1).

Based on the list of potential targets, Roche Sequencing Solutions designed a list of candidate baits. Exon–intron boundaries were identified by mapping Roche‐designed target areas to the unmasked *Pinus taeda* reference genome v2.01 (Zimin et al. 2017) using blastn (Altschul et al. 1990). Since *P. sylvestris* does not have a public reference genome, *P. taeda* reference was used in the mapping, as it was the highest quality reference genome available from a related species at the time of bait set design. The mapping process was parallelised using the workflow management software STAPLER (Tyrmi 2018). Exon‐intron boundary was inferred based on alignments where the best alignment did not span the whole target length (https://github.com/tyrmi/PGU/tree/master/find_exon_boundaries). Baits overlapping with any of the 10,748 identified exon–intron boundaries or mapping to highly repetitive regions were excluded from the design. Baits were allowed up to 20 close matches to the reference genome. The final bait set design: SeqCap Design PiSy_UOULU (File S2) covers 18,516 regions with a total capture space of 9,345,488 bp.

### 2.2 DNA Extractions and Library Preparations

A single *P. sylvestris* genotype (E1101), chosen based on the availability of additional sequencing data from the same individual, was used in the exome captures to avoid the effects of genetic variation between different individuals. Before each DNA extraction, 100 mg of frozen needles were cut into < 5 mm pieces and disrupted using TissueLyser (Qiagen) at 30 1/s for 2 × 120 s. The disrupted samples were incubated in SP1 buffer at 65°C for one hour, followed by DNA extraction using E.Z.N.A SP Plant DNA Kit (Omega Bio‐tek). To increase DNA yield, two additional steps were performed: NaOH treatment of the column before loading the sample and double elution to the same supernatant. DNA concentration was measured using Quant‐iT PicoGreen dsDNA kit (Thermo Fisher Scientific).

DNA extraction was followed by DNA fragmentation. Samples were first diluted to 20 ng/μL concentration using TE buffer (1 mM Tris–HCl, pH 8.0, 0.01 mM EDTA). Sample DNA was then fragmented using Bioruptor UCD‐200 (Diagenode) for 2 × 15 min + 13 min in 30/90 s on/off‐cycles with power setting ‘L’. DNA fragmentation was followed by double‐sided size selection using Agencourt AMPure XP beads (Beckman Coulter) to extract fragments in the desired 250–450 bp range. Beads to sample volume ratio of 0.7 was used for the left side selection and 0.125 for the right side selection. The fragment size distribution was quantified using Bioanalyzer 2100 (Agilent Technologies) with Agilent DNA 12000 Kit (Agilent Technologies).

Libraries were prepared using Kapa HyperPrep Kit (Kapa Biosystems) with SeqCap Adapter Kit (Roche). Ampure XP beads were used in all of the bead‐based clean‐up steps. DNA concentrations were measured (Quant‐iT PicoGreen dsDNA Kit) before the amplification step to determine the minimal number of required PCR cycles. All libraries underwent three cycles of PCR. DNA fragment size distribution for libraries was analysed using Bioanalyzer 2100 with Agilent High Sensitivity DNA Kit, and DNA concentrations were measured using Quant‐iT PicoGreen dsDNA Kit.

### 2.3 C0t‐1 DNA Preparation

Species‐specific c0t‐1 DNA was prepared for *P. sylvestris* according to the protocol published by Zwick et al. (1997) with the following modifications. (1) DNA was fragmented using Bioruptor UCD‐200 (1 × 10 min + 1 × 6 min, 30/90 s on/off‐cycles, power setting ‘M’) instead of using an autoclave. (2) The targeted fragment size distribution was lowered from the 100–1000 bp range to the 100–500 bp range (peak between 300 and 500 bp) to match the size of our library fragments better. Fragmented DNA was diluted to 500–600 ng/μL concentration instead of the recommended 100–500 ng/μL concentration due to the high DNA concentration of our samples and to keep the total reaction volume low. (3) The activity of S1 Nuclease was stopped by adding EDTA (0.5 M, pH 8.0) and incubating at +70°C for 10 min instead of the suggested phenol extraction, as it is easier and safer to perform with EDTA. (4) The DNA pellet was washed with 70% ethanol three times after the overnight precipitation, instead of just spinning down the pellet. (5) The DNA pellet was dissolved in 10–20 μL of Tris–HCl (10 mM, pH 8.0) instead of 100–200 μL of TE to minimise the volume of c0t‐1 DNA added to the hybridisation reaction of exome captures.

The main steps of the complete modified protocol are described here briefly, for the full version see File S3. DNA was extracted from fresh *P. sylvestris* needles using E.Z.N.A SP Plant DNA Kit and fragmented using Bioruptor UCD‐200. DNA was fragmented to < 500 bp long fragments, confirmed by agarose gel electrophoresis (1% agarose gel, 100 V, 30–40 min). Fragmented DNA was diluted to 500–600 ng/μL DNA concentration and 0.3 M NaCl concentration using 5 M NaCl and PCR‐grade water. NaCl is used to induce breaks in double‐stranded DNA. Diluted DNA was incubated at 95°C for 10 min to denature it. Denatured DNA was allowed to reanneal by incubating at 65°C for ~11 min. Incubation time for the reannealing reaction was calculated using the following formula: *C*_*o*_ *t* = 1 = mol/L × *T*_*s*_, where *C*_*o*_ is nucleotides per litre and *T*_s_ is time in seconds (Zwick et al. 1997). Repetitive DNA sequences reanneal faster due to their higher probability of finding complementary strands. The remaining single‐stranded DNA molecules (non‐repetitive DNA) were digested by adding S1 nuclease (Thermo Fisher Scientific) and incubating at +37°C for 15 min. The activity of S1 nuclease was stopped by adding EDTA (0.5 M, pH 8.0) and incubating at +70°C for 10 min. Glycogen (Thermo Fisher Scientific), sodium acetate and 100% ethanol were added to the DNA solution. The DNA solution was stored at −80°C overnight. DNA was precipitated on the following day. The cleaned DNA pellet was dissolved in 10 μL Tris–HCl (10 mM, pH 8.0). C0t‐1 DNA purity and concentration were measured using Nanodrop (Thermo Fisher Scientific). Multiple c0t‐1 DNA extracts were combined to create pools of 30,000 and 60,000 ng.

### 2.4 Exome Captures

Six exome captures were performed with three different treatments and two replicates per treatment for blocking the binding of repetitive DNA sequences to the capture baits and to capture‐bound genomic DNA fragments. Two of the treatments were conducted using our in‐house *P. sylvestris* c0t‐1 DNA in two different amounts: 30,000 ng or 60,000 ng. The third treatment was conducted using 10 μL of the commercial blocker SeqCap EZ Developer Reagent (Roche). The concentration and contents of the Developer Reagent are not disclosed by Roche. The exome captures were performed using a SeqCap EZ HyperCap (Roche) kit with the custom bait set. DNA input for all exome captures was 1000 ng and the incubation time for the hybridisation reaction was 20 h. DNA fragment size distribution for the captured libraries was analysed using Bioanalyzer 2100 with Agilent High Sensitivity DNA Kit. DNA concentrations were measured using Quant‐iT PicoGreen dsDNA Kit. Captured libraries were quantified using LightCycler 480 (Roche) with Kapa Library Quant Kit (Roche) and diluted to 10 nM concentration before sequencing. Sequencing was performed at Biocenter Oulu Sequencing Center using NextSeq 550 System (Illumina) with NextSeq 500/50 Mid Output v2.5 kit (300 cycles).

### 2.5 Whole‐Genome Sequencing

Whole‐genome sequencing was conducted to estimate how much repetitive DNA is being sequenced when no capture and blocking treatments are applied. DNA used in WGS was extracted from the haploid megagametophyte tissue from seeds of the same genotype (E1101) that was used in the exome captures. Seed germination was induced by placing seeds onto a moist filter paper on a Petri dish and incubating at room temperature overnight. Germinating seeds were then dissected under a microscope to separate the seed coat, megagametophyte and embryo. DNA extraction, fragmentation, size selection and quantification were performed in the same way as described in the section ‘*DNA extractions and library preparations*’. Library preparation was conducted with NEBNext Library Prep Master Mix Set for Illumina (New England Biolabs) with NEBNext Multiplex Oligos for Illumina (New England Biolabs). Ampure XP beads were used in all the bead‐based cleanup steps. DNA fragment size distribution of the library was analysed using Bioanalyzer 2100 with Agilent High Sensitivity DNA Kit and DNA concentration was measured using Quant‐iT PicoGreen dsDNA Kit. The library was sequenced at the Institute of Molecular Medicine Finland (FIMM) using HiSeq 2500 System (Illumina).

### 2.6 Read Mapping

De‐multiplexing and adapter trimming for the raw exome capture sequencing data were performed by Biocenter Oulu Sequencing Center and for the whole‐genome sequencing data by FIMM. Raw read quality for each sample was analysed using FastQC v0:11.8 (Andrews 2010). FastQC results were aggregated using MultiQC v1.9 (Ewels et al. 2016) and then compared to each other.

*P. sylvestris* does not have a public reference genome available. *P. taeda* reference (v2.01) genome was used for mapping during the bait set design steps. However, the first chromosome level conifer reference genome was recently released for the *P. tabuliformis* (Niu et al. 2022), which is a closer relative to *P. sylvestris* than *P. taeda* (Jin et al. 2021). A masked version of *P. tabuliformis* reference genome (available on Dryad) was used as the reference genome in all of the following bioinformatic analyses. Masking was performed to increase unique mapping to the genome by correcting problems in genome polishing, as many identical sequences were found at the ends of different chromosomes.

To construct a masked version of the reference genome, chromosomes were first split back into contigs. Contigs were then aligned within chromosomes and between unplaced contigs using Minimap2 (Li 2018a). Alignments were then chained to longer ones using the ChainPaf module of Lep‐Anchor (Rastas 2020). Half of the aligning regions of > 10 kb were masked by masking the region in the shorter of the two contigs involved in the alignment. In total, 7,598,047,035 (29.9%) bases were masked out of the 25,421,342,128 bases in the reference. The masked reference was indexed using bwa (v.0.7.17; Li and Durbin 2009) index command.

Raw exome capture reads were mapped to the indexed reference using bwa‐mem algorithm with default parameters. The resulting SAM files were converted to BAM files, sorted and de‐duplicated using SAMtools v1.9 (Danecek et al. 2021) commands sort, fixmate and markdup (−r). Read group tags were added using Picard tools v.2.21.4 (Picard Toolkit 2019) AddOrReplaceReadGroups command. To incorporate the variation in the number of mapped reads between different samples into the analyses, a random subsample of 45 million reads was taken from all de‐duplicated BAM files using SAMtools view command (−s 45,000,000/number of mapped reads in the sample). Subsampled BAM files were indexed using SAMtools index command with the option ‐c (CSI index). Regular BAI index format was not used, as it only supports individual chromosomes up to 512 Mbp in length.

### 2.7 Coverage Analysis

The regions targeted by exome capture baits were based on *P. sylvestris* transcriptome data. These transcripts were mapped to the masked *P. tabuliformis* reference using the bwa‐mem algorithm with default parameters and converted to a BAM file using the SAMtools view command. Mapped transcripts were then filtered based on the mapping quality (MAPQ > 20) using SAMtools view command (−q 20). The filtered BAM file was then converted to a BED file using Bedtools v2.27.1 (Quinlan and Hall 2010) bamtobed command. Mapped transcripts were annotated to the *P. tabuliformis* reference genome v1.0 annotation file modified for the masked genome (Dryad) using the Bedtools intersect command to determine whether they were located within annotated genes. Annotation was done to estimate the accuracy of mapping, as genic regions are assumed to be highly similar between the two species due to shared evolutionary history (Jin et al. 2021).

The BED file for mapped transcripts was converted to a Picard Interval List using the Picard tools BedToIntervalList command. Coverage and various other statistics were collected from all subsampled exome capture BAM files using Picard tools CollectHsMetrics command. The Picard Interval list was used as both the target intervals and bait intervals list in the command because we do not have the sequences for the baits. The same statistics were also collected for the whole annotated exome of *P. tabuliformis* . This was done by first removing targeted regions from the list of annotated exons using the Bedtools subtract command, converting the resulting BED file to a Picard Interval List and then running Picard CollectHsMetrics for all subsampled exome capture BAM files. To estimate sequencing coverage variation on a broader scale, a random set of 100,000 intervals with a length of 100 bp was generated from the masked *P. tabuliformis* reference using the Bedtools random command (−n 100,000). Coverage for these random intervals was calculated within each sample using the Bedtools coverage command.

### 2.8 Variant Calling

A joint variant call for the subsampled exome capture BAM files was performed using BCFtools v1.9 (Danecek et al. 2021) with mpileup and call commands (bcftools mpileup ‐Ou ‐‐annotate AD, DP ‐f masked.reference ‐‐bam‐list | bcftools call ‐vmO z ‐o output.vcf.gz). Variant calling was used as a tool for comparing the performance of different exome capture treatments. Because only a single genotype was used in the exome captures, all genotype calls were expected to be identical between the different treatments. SNPs (−i ‘TYPE=“snp”’) originating from the differences between sequences of *P. sylvestris* and *P. tabuliformis* (identified by 1/1‐homozygous genotype calls) were removed (−e ‘COUNT(GT=“AA”)=N_SAMPLES || COUNT(GT=“RR”)=N_SAMPLES’). SNPs containing the same genotype calls across all treatments were removed (−e ‘AC == AN’), as we were interested in SNPs that had different genotype calls between the treatments. The remaining SNPs were filtered based on the following criteria: SNP quality > 30, total depth > 30 and mapping quality > 50 (−i ‘QUAL > 30 && INFO/DP > 30 && MQ > 50'). All of the above‐mentioned filtering steps were performed using the BCFtools view command. VCFtools v0.1.17 (Danecek et al. 2011) ‐‐minDP filter was used to set all genotypes that had less than five reads supporting them, as missing. The total number of missing genotype calls per sample was calculated using VCFtools ‐‐missing‐indv command. VCF file containing the filtered SNPs was converted to a BED file using BEDOPS v2.4.41 (Neph et al. 2012) vcf2bed command. The number of SNPs was calculated using VCFtools ‐‐bed and ‐‐exclude‐bed commands to generate separate VCF files for the on‐target and off‐target SNPs. The off‐target VCF file was annotated to the *P. tabuliformis* reference genome v1.0 annotation file modified for the masked genome (Dryad) using the Bedtools intersect command to determine whether they were located within annotated genes. Bedtools slop (−b) was then used to extend the annotated genes by 10,000 bp in both directions to count the number of SNPs in nearby genes.

### 2.9 Tandem Repeats

The whole‐genome sequencing and exome capture reads were converted from fastq to fasta format using Seqtk v.1.3‐r106 (Li 2018b) seq command. Tandem Repeats Finder v.4.09.1‐1 (Benson 1999) with the recommended parameters (matching weight 2, mismatch penalty 5, indel penalty 7, match probability 80, indel probability 10, minimum alignment score 50 and a maximum period size 2000) was used to identify tandem repeats from the WGS data (total of 516,799,922 reads). The occurrence of each identified tandem repeat was counted. These counts gave information about the most common tandem repeats in the *P. sylvestris* genome, as no treatment was used for blocking the sequencing of repetitive DNA sequences for the WGS. Raw reads from exome capture and WGS were then randomly subsampled to 51 million reads to match the sample with the smallest number of reads (30K_B) using the Seqtk sample command (−s 10) to allow equal comparisons. Tandem Repeats Finder was then used with the recommended parameters to identify tandem repeats from all the subsampled samples. Tandem repeat counts from the subsampled samples were then compared to each other for the most common tandem repeats found from the first run of Tandem Repeats Finder with all of the WGS data.

**FIGURE 1:**
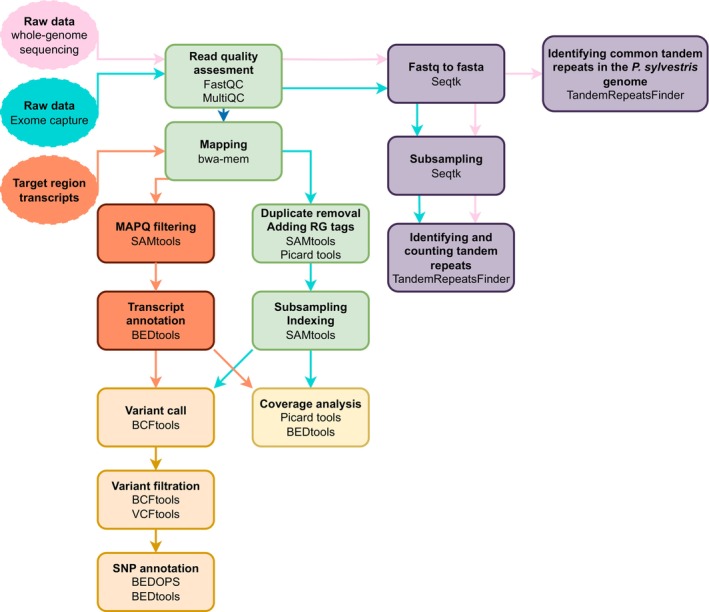
Overview of the bioinformatics pipeline.

The flow of all the above‐mentioned bioinformatics steps and their relations to each other has been described in Figure 1.

## 3 Results

### 3.1 Sequencing Results and Bait Set Performance

We generated 53.94 Gbp of 150 bp paired‐end sequencing data from six exome captures (three different treatments and two replicates of each treatment) using a novel bait set. The number of raw reads per exome capture varied between 52,865,561 and 64,657,074 (Table 1). Mapping rates were high, over 99% of reads were mapped in all of the treatments. The 60,000 ng c0t‐1 DNA treatment had the highest percentage of mapped reads 99.27%–99.30% and the lowest percentage of duplicate reads 10.11%–10.97% (Table 1). The Developer Reagent had the largest percentage of duplicated reads. However, the overall differences between the treatments in these aspects were small.

**TABLE 1:** Exome capture read and mapping statistics for raw sequencing reads.

Sample	Raw reads	Duplicate reads	Mapped reads
60K_A	58,078,241	6,369,885 (10.97%)	51,435,497 (99.27%)
60K_B	57,104,428	5,772,540 (10.11%)	51,076,900 (99.30%)
30K_A	55,188,928	7,483,552 (13.56%)	47,412,572 (99.18%)
30K_B	52,865,561	6,385,977 (12.08%)	46,571,837 (99.26%)
DR_A	58,235,220	8,309,142 (14.27%)	49,627,974 (99.22%)
DR_B	64,657,074	8,118,184 (12.56%)	56,130,067 (99.10%)

*Note:* 60K = 60,000 ng species‐specific c0t‐1 DNA treatment, 30K = 30,000 ng species‐specific c0t‐1 DNA treatment and DR = Developer Reagent treatment (universal blocker). A and B are treatment replicate identifiers.

By mapping, we successfully identified 18,460 out of 18,516 *P. sylvestris* target regions in the *P. tabuliformis* reference, of which 16,060 were retained after filtering based on mapping quality. Filtered target regions were annotated using the *P. tabuliformis* reference genome annotations. Of the 16,060 filtered target regions, 14,037 (87.4%) were located within annotated genes. In conclusion, target regions were reliably located within the *P. tabuliformis* reference, as they were mapped with high confidence and the majority resided within annotated genes.

### 3.2 Sequencing Coverage

**FIGURE 2:**
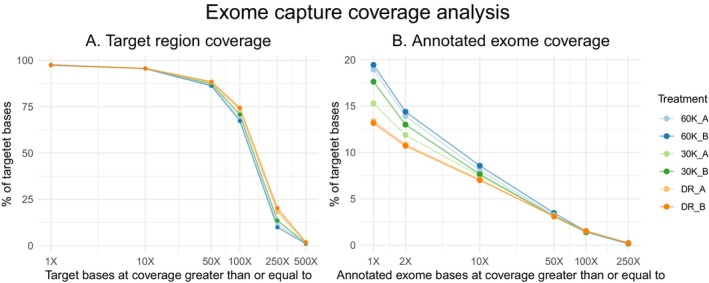
Sequencing coverage for (A) the target regions and (B) the annotated exome.

Sequencing coverage for all of the target regions was analysed in all of the exome captures. The majority of the target regions were fully covered in all of the treatments, as close to 100% of the targeted bases were covered by at least one read (Figure 2A). The mean target coverage varied between 147.7× (60,000 ng c0t‐1 DNA treatment) and 178.6× (Developer Reagent treatment). The Developer Reagent treatment produced higher coverage for over 80% of the targeted bases (Figure 2A) and it had the highest percentage of bases on target (28.3%–29.2%). Despite the developer reagent treatment having the highest mean coverage, it also had the highest number of zero coverage targets (Table 2) and fewer fully covered target regions compared to both of the c0t‐1 DNA treatments (Figure S1). It also had noticeably higher maximum target coverage compared to the 60,000 ng c0t‐1 DNA treatment (Table 2). The higher mean coverage and high max target coverage indicate that the Developer Reagent captured more copies of the same DNA fragments. The two replicates of the 30,000 ng c0t‐1 DNA treatments clearly separated from each other, replicate (A) being more similar with the Developer Reagent treatment and replicate (B) being more similar with the 60,000 ng c0t‐1 DNA treatment.

**TABLE 2:** Coverage statistics for the target regions.

Sample	% Bases on‐target^a^	Max target coverage	Mean target coverage	% Zero coverage targets
60K_A	25.12	4325	153.22	3.75
60K_B	24.21	4010	147.74	3.63
30K_A	28.70	5283	175.59	3.81
30K_B	26.25	4308	160.29	3.67
DR_A	28.30	5449	172.81	4.05
DR_B	29.20	5528	178.63	4.04

^a^ The number of aligned, de‐duplicated, on‐target bases out of all the bases available.

Sequencing coverage was also analysed for the annotated exome of *P. tabuliformis* to estimate how much of the off‐target capture consists of annotated exons. The *P. tabuliformis* exome consists of 624,800 exons in 80,491 genes with a total area of ~232.5 Mbp. The 60,000 ng c0t‐1 DNA treatment produced the highest percentage of bases in the coverage class greater than or equal to one (Figure 2B). The Developer Reagent had the smallest number of bases at this coverage and the same trend continued until the 50× coverage value. These results show that the 60,000 ng c0t‐1 DNA treatment captured a wider range of exons from the *P. tabuliformis* exome compared to the developer reagent and to the 30,000 ng c0t‐1 DNA treatment. Both c0t‐1 DNA treatments outperformed the Developer Reagent. The 30,000 ng c0t‐1 DNA treatment (replicate A) was clearly separated from the coverage produced by the Developer Reagent, unlike in the target regions. However, the calculated coverage values between the treatments do not highlight these differences, as the percentage of bases on‐exome and mean exome coverage are highly similar to each other (Table 3). This is due to the presence of exons with extremely high sequencing coverage, which are hiding the differences observed in the breadth of coverage (Figure 2B). Only around 10% of all sequenced reads were mapped to the exome (in addition to 87.4% of target regions being mapped to annotated genes and containing 24%–29% of the sequenced reads), leaving large parts of the off‐target capture unaccounted for. This is likely due to differences in the locations of exons and the lengths of introns between the two species.

**TABLE 3:** *P. tabuliformis* exome coverage statistics. Target regions located within the exome are excluded.

Sample	% Bases on exome^a^	Max exon coverage	Mean exon coverage	% Zero coverage exons
60K_A	9.44	6813	6.04	29.96
60K_B	9.87	6549	6.32	29.69
30K_A	9.73	8208	6.25	31.75
30K_B	9.15	7241	5.87	30.49
DR_A	9.40	8851	6.02	32.73
DR_B	9.44	9279	6.06	32.84

^a^ The number of aligned, de‐duplicated, on‐exome bases out of all the bases available.

### 3.3 Variant Calling

Variant calling was used as a tool for comparing the performance of different exome capture treatments to each other. All exome captures were conducted using DNA from the same genotype, and therefore all genotype calls were expected to be identical across the treatments. A total of 43,955,983 variants were detected (SNPs + indels), of which a set of 535,973 SNPs remained after filtering. Less than 15% of the detected SNPs resided within the target regions (69,278). The rest of the SNPs (466,695) resided outside the target regions, of which 29.3% were located within annotated *P. tabuliformis* genes and 44.1% within 10,000 bp of an annotated gene, indicating that a large part of the off‐target capture also consisted of exons.

**FIGURE 3:**
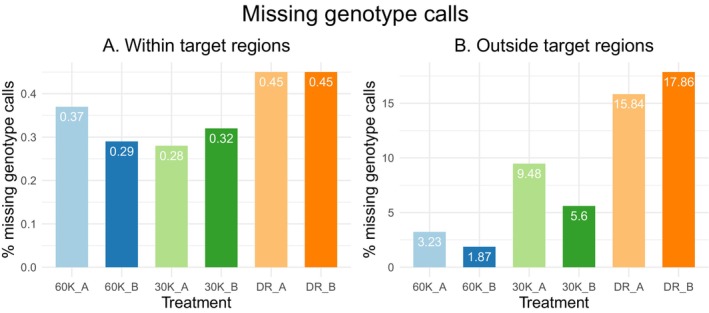
The percentage of missing genotype calls for SNPs detected (A) within target regions (69,278 SNPs in total) and (B) outside target regions (466,695 SNPs in total).

The small portion of SNPs being located within the target regions compared to the off‐target regions implies that target regions had enough sequencing depth in all treatments to produce confident genotype calls. This was confirmed by comparing the percentages of missing genotype calls between the treatments for SNPs found within target regions (Figure 3A). The percentage of missing genotype calls was low for all treatments, and it varied between 0.28% and 0.45%. The Developer Reagent treatment produced slightly more missing genotype calls in the target regions compared to both c0t‐1 DNA treatments even though it had the highest median coverage for these regions. Striking differences were observed when the percentages of missing genotype calls were compared for the SNPs found outside the target regions (Figure 3B). The Developer Reagent had by far the largest percentage of missing genotype calls (15.8%–17.9%). The 60,000 ng c0t‐1 DNA treatment had only between 1.87% and 3.23% missing genotype calls, which is around six and half times less than the Developer Reagent produced. The 30,000 ng c0t‐1 DNA treatment produced between 5.6% and 9.48% missing genotype calls, placing it between the other two treatments. These results are in line with the results from coverage analysis and show that exome captures with species‐specific c0t‐1 DNA blockers are able to capture more unique sequences (exons) within the target regions and especially outside the targeted regions, and therefore produce more SNPs than exome captures with the Developer Reagent blocker. Doubling the amount of the c0t‐1 DNA in exome capture leads to increased capture of unique sequences.

### 3.4 Tandem Repeats

**FIGURE 4:**
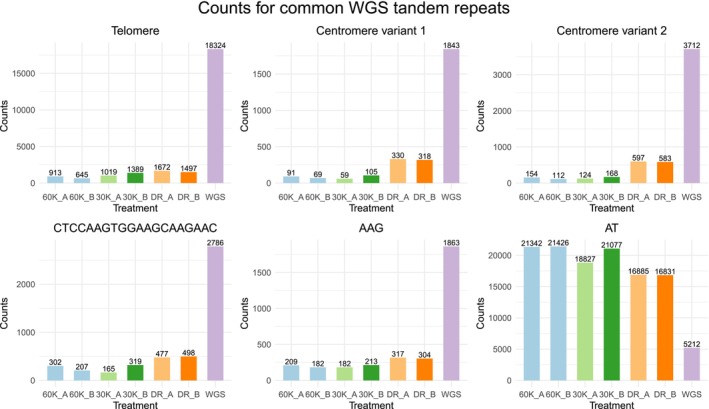
Counts for some of the most common tandem repeats identified in the Whole‐genome sequencing data. Telomere repeat contains counts for telomere sequence TTTAGGG and the following variants identified by TRF: TTAGGGT, GTTTAGG, TAGGGTT and GGTTTAG. Centromere variant 1 is TGGAAACCCCAAATTTTGGGCGCCGGG and centromere variant 2 is TGGAAACCCCAAATTTTGGGCGCCGCA.

Whole‐genome sequencing conducted without any treatment for blocking the sequencing of repetitive DNA was used to identify common tandem repeats in the *P. sylvestris* genome (File S4). Counts for some of the most common tandem repeats were compared to the different exome capture treatments (Figure 4). WGS produced a substantially higher number of copies of the telomere repeat TTTAGGG and its different variants compared to the exome captures. Similar observations were made for the two centromere repeat variants, repeat motif CTCCAAGTGGAAGCAAGAAC, and trinucleotide repeat AAG (shown in Figure 4). Both of the c0t‐1 DNA treatments produced fewer copies of all of these tandem repeats compared to the Developer Reagent treatment, demonstrating that the use of the species‐specific c0t‐1 DNA reduces sequencing of common tandem repeats, such as telomeres and centromeres.

**FIGURE 5:**
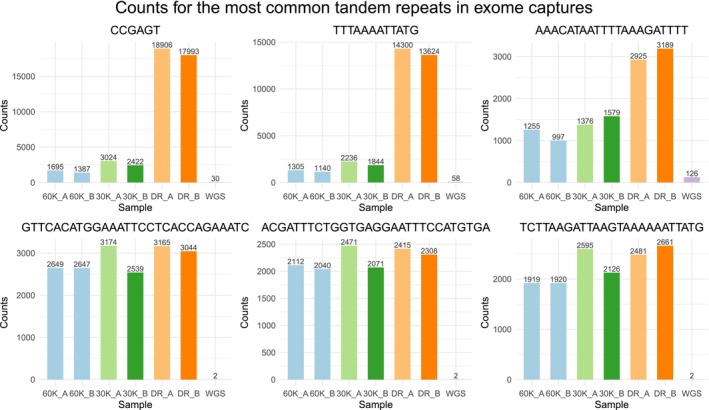
Counts for the most common tandem repeats (longer than 3 bp) found from Developer Reagent treatments and c0t‐1 DNA treatments. Tandem repeats in the upper row are the most common tandem repeats found in the Developer Reagent treatment and tandem repeats in the lower row are the most common tandem repeats found in both of the c0t‐1 DNA treatments.

The mono‐ and dinucleotide repeats (A)n, (T)n, (G)n and (AT)n were the most common types of tandem repeats found in both of the c0t‐1 DNA treatments and among the most common tandem repeats in the Developer Reagent treatment (File S4). Repeat motif (AT)n was three to four times more common in the exome captures than in the WGS (Figure 4). The high number of these types of repeats indicates that they are located within or close to genes. Counts for the most common tandem repeats in each of the exome capture treatment were compared to each other and to the WGS. The three most common tandem repeats (excluding all mono‐, di‐ and trinucleotide repeats) found from the Developer Reagent treatment had considerably more copies in Developer Reagent compared to both of the c0t‐1 DNA treatments (Figure 5, upper row). Repeat motifs (CCGAGT)n and (TTTAAAATTATG)n had over ten times more copies in the Developer Reagent treatment compared to the 60,000 ng c0t‐1 DNA treatment. Developer Reagent treatment also produced more copies of the three most common tandem repeats (excluding all mono‐, di‐ and trinucleotide repeats) shared by both c0t‐1 DNA treatments (Figure 5, bottom row) compared to the 60,000 ng c0t‐1 DNA treatment. Results from the tandem repeat analysis show that the use of the species‐specific c0t‐1 DNA blocker reduces the sequencing of the common tandem repeats found in the genome and also the sequencing of tandem repeats more specific to exome captures compared to Developer Reagent blocker. The differences between the c0t‐1 DNA treatments are rather small and lack any clear trends, indicating that using at least 30,000 ng of c0t‐1 DNA per exome capture is generally enough to reduce the sequencing of tandem repeats.

## 4 Discussion

The main goal of this study was to compare three different treatments in exome captures for blocking the binding of repetitive DNA sequences to the capture baits and the capture‐bound genomic DNA fragments in *P. sylvestris* , a species with a large and highly repetitive genome. One of the treatments used a commercially available universal blocker for plants (Developer Reagent) and two of the treatments used species‐specific c0t‐1 DNA in two different quantities: 30,000 and 60,000 ng. We designed a custom bait set consisting of 18,516 target regions and used it in the exome captures to compare these different treatments to each other. The largest differences between species‐specific c0t‐1 DNA treatments and the universal blocker treatment were observed in the amounts of ‘off‐target’ capture. The 60,000 ng c0t‐1 DNA treatment produced sequencing coverage for the largest portion of exons in the annotated exome of *P. tabuliformis* . It also had by far the smallest amount of missing genotype calls for the SNPs found in the off‐target regions, meaning that it had the largest amount of sequencing reads from these regions to produce confident genotype calls. The 30,000 ng c0t‐1 DNA also outperformed the universal blocker in these aspects. Exome capture bait sets target more exons and other sequences than the initial set of targets due to the non‐specificity of the baits. This is a generally desirable feature, as bait sets are usually not broad enough to target the whole exome of the species. Capture baits used in this experiment were allowed up to 20 close matches to the reference genome ( *P. taeda* v2.01). It is notable that the extent of off‐target exome capture was quite large, as the 60,000 ng c0t‐1 DNA treatment produced coverage for close to 20% of the annotated exome. The total size of the annotated exons with targeted regions excluded was ~212 Mbp, meaning that the captured off‐exon capture accounted for 42.4 Mbp. This is over four times larger area than our original ~9.3 Mbp target. However, it is noteworthy that over 91.2% of genes in the *P. tabuliformis* were identified as being duplicated through dispersed duplication (Niu et al. 2022). Reads mapping to the wrong duplicates of the genes can make the off‐target capture appear to be more extensive than it is in reality.

Differences between the treatments were also observed in the amounts of captured tandem repeats. Both of the species‐specific c0t‐1 DNA treatments captured fewer copies of some of the most common tandem repeats found in the *P. sylvestris* genome, such as the telomere and the centromere repeats, compared to the universal blocker treatment. They also captured fewer copies of the exome capture specific tandem repeats. The differences between the c0t‐1 DNA treatments in capturing tandem repeats were small, suggesting that the use of species‐specific c0t‐1 DNA instead of a universal blocker is a more important factor than the amount of c0t‐1 DNA used. Exome captures produced more copies of mono‐ and dinucleotide repeats (A)n, (T)n, (G)n and (AT)n compared to the whole‐genome sequencing. The high copy numbers of (AT)n repeat motif in exome captures could be partially explained by the sequencing of TATA boxes located close to transcription start sites. The mononucleotide repeat (A)n and many other short tandem repeats have been linked to *cis*‐regulatory elements in gene expression regulation in eukaryotes (Horton et al. 2023; Pholtaisong et al. 2022), explaining their abundance in exome captures. However, the abundance of poly‐G repeats is a common artefact in two‐channel Illumina sequencing systems observed when the dark base G is called after the termination of synthesis. Interestingly, many of the most common tandem repeats found in the universal blocker treatment were in multiples of three, suggesting they could be potential amino acid repeats. The high number of sequenced amino acid repeats can be a sign of a less diverse sequencing library, which is in line with our findings from sequencing coverage and variant call analyses.

Targeted regions were highly covered in all treatments. Differences between the species‐specific c0t‐1 DNA treatments and the universal blocker treatment were small. The universal blocker treatment had the highest number of reads mapping to the targeted regions and had the highest coverage. However, there was still somewhat higher percentage of zero coverage targets and fewer targets with full sequencing coverage, meaning that a larger part of the capture consisted of the same fragments sequenced repeatedly in the universal blocker treatment. Both of the species‐specific c0t‐1 DNA treatments produced more than enough coverage for the target regions while providing more reads from off‐target exons. The higher number of captured off‐target exons allows the discovery of more SNPs and indels. This, combined with the reduced capture of various tandem repeats, makes it easy to recommend the use of species‐specific c0t‐1 DNA in exome captures. Based on our results, we recommend using at least 60,000 ng of species‐specific c0t‐1 DNA in exome captures instead of the universal blocker. C0t‐1 DNA can be prepared with a short two‐day protocol relatively cheaply, as it does not require any highly specialised equipment or expensive reagents. The lower 30,000 ng of c0t‐1 DNA treatment was based on the results by McCartney‐Melstad, Mount, and Shaffer (2016), who suggested using at least 30,000 ng of c0t‐1 DNA per 1000 ng of input DNA. They found that increasing the amount of c0t‐1 DNA increased the percentage of unique reads mapping to target regions in amphibian exome captures (McCartney‐Melstad, Mount, and Shaffer 2016). Doubling the amount of species‐specific c0t‐1 DNA to 60,000 ng in exome captures led to higher amounts of off‐target capture from exons with no negative side effects. We speculate that even higher quantities could be beneficial. However, more testing with higher quantities of c0t‐1 DNA and different multiplexing strategies would be required. More replicates would also be required to fully understand the variation within the treatments and to differentiate random fluctuations from true effects. The 30,000 ng c0t‐1 DNA treatment had a lot of within‐treatment variation due to the subpar performance of one of the replicates (A) in the hybridisation reaction.

C0t‐1 DNA has some interesting applications outside of its use in blocking the binding of repetitive DNA sequences in exome captures. It can be used in fluorescence in situ hybridisation (FISH) to locate repetitive DNA sequences within chromosomes (Chang et al. 2008; Sevilleno et al. 2020) or it can be sequenced to analyse the repetitive DNA (low c0t DNA) contents within a genome. This approach has been used to analyse repetitive DNA contents within chicken (Wicker et al. 2005), banana (Hřibová et al. 2008) and beet (Zakrzewski et al. 2010) genomes. Fragment sizes of c0t‐1 DNA can be easily adjusted to match the fragment sizes required by the selected sequencing method. Different reannealing times can be used to either only target the highly repetitive DNA sequences or also some moderately repetitive DNA sequences. Repetitive sequence motifs within tandem repeats and interspersed repeats are typically rather short, meaning that regular short‐read sequencing methods, such as Illumina sequencing, can be used.

An even more interesting application is the use of high c0t DNA (low copy number DNA) in whole‐genome sequencing. Different DNA re‐association times can be used to separate repetitive DNA sequences from unique DNA sequences (genes). After certain reassociation time, repetitive DNA sequences are in a double‐stranded form and unique DNA sequences are in a single‐stranded form. The single‐stranded DNA can then be separated from the double‐stranded DNA by using hydroxyapatite chromatography (HAP chromatography) (Peterson et al. 2002) or by using duplex‐specific nuclease to hydrolyse double‐stranded DNA (Shagina et al. 2010). Peterson et al. (2002) suggested using HAP chromatography to separate single‐stranded DNA from double‐stranded DNA after renaturation to specific c0t values to efficiently capture unique sequences from eukaryote genomes. A method based on the same idea of using DNA renaturation to normalise repetitive DNA was developed and successfully tested with maize (Yuan, SanMiguel, and Bennetzen 2003). Shagina et al. (2010) suggested using duplex‐specific nuclease extracted from Kamchatka crab to eliminate double‐stranded low c0t DNA, as an alternative to using HAP chromatography. Both methods offer powerful ways to eliminate repetitive DNA sequences from whole‐genome sequencing data sets and are a great alternative to currently popular targeted sequencing methods. As opposed to exome capture, they do not require any prior knowledge about the genes nor are as random as restriction‐based methods. These methods appear having been largely forgotten during the rapid advances in sequencing technologies within the 2000s and should now be revisited.

## 5 Conclusions

We demonstrated that exome capture can be further optimised for species with large and highly repetitive genomes by using species‐specific c0t‐1 DNA to block the binding of repetitive DNA sequences to the capture baits and the capture‐bound genomic DNA fragments during the hybridisation reaction. This approach can be used with all existing bait set designs and c0t‐1 DNA can be prepared following a short two‐day protocol without expensive reagents. Based on our results, we recommend using at least 60,000 ng of species‐specific c0t‐1 DNA for 1000 ng of input DNA in exome captures, as larger quantities of it were observed to produce more unique reads from exons, especially in the ‘off‐target’ regions.

## Author Contributions

Tanja Pyhäjärvi, Outi Savolainen, Sonja T. Kujala, Jaakko S. Tyrmi and Tiina M. Mattila designed the bait set. Timo A. Kumpula and Robert Kesälahti optimised the protocol for producing species‐specific c0t‐1 DNA for *P. sylvestris* . Robert Kesälahti, Sandra Cervantes and Tanja Pyhäjärvi designed the experiment. Robert Kesälahti, Alina K. Niskanen and Tanja Pyhäjärvi analysed the data. Pasi Rastas created the masked version of *P. tabuliformis* reference genome. Robert Kesälahti wrote the manuscript with the help of Alina K. Niskanen and Tanja Pyhäjärvi.

## Conflicts of Interest

The authors declare no conflicts of interest.

### Open Research Badges

This article has earned an Open Data badge for making publicly available the digitally‐shareable data necessary to reproduce the reported results. The data is available at https://www.ncbi.nlm.nih.gov/bioproject?LinkName=sra_bioproject&from_uid=34651786 and https://doi.org/10.5061/dryad.qfttdz0rw.

## Supporting information


**FILE S1.** Transcript IDs (Ojeda et al. 2019) for the 2435 candidate genes for phenology and for primary and secondary metabolism pathways active during heartwood formation.

**FILE S2.** Fasta sequences for the 18,516 targeted *P. sylvestris* regions.

**FILE S3.** Laboratory protocol for preparing c0t‐1 DNA from plant material (optimised for conifers).

**FILE S4.** Counts for the identified tandem repeats from all treatments and WGS data.

**FIGURE S1.** Sequencing coverage for target regions plotted as a kernel density estimate (KDE) plot.

## Data Availability

Raw sequence reads are deposited in the SRA (BioProject PRJNA1145653). The masked *P. tabuliformis* reference genome and annotations with updated genomic coordinates are available on Dryad (doi:10.5061/dryad.qfttdz0rw).
