# Supplementary material for: Optimising Exome Captures in Species With Large Genomes Using Species‐Specific Repetitive DNA Blocker
Source: Mol Ecol Resour. 2024 Dec 18;25(3):e14053. doi: 10.1111/1755-0998.14053 (PMC11887611; doi:10.1111/1755-0998.14053)
Supplement: Supplementary file 3 — FILE S3. Laboratory protocol for preparing c0t‐1 DNA from plant material (optimised for conifers). [file MEN-25-e14053-s003.pdf]

C0t-1 DNA Protocol for Plants

Version 1.1

Timo Kumpula & Robert Kesälahti

9/2024

Contents

1 Introduction **2**

2 Required Materials **2**
2.1 Equipment 2
2.2 Reagents and Consumables 2

3 DNA extraction **3**
3.1 Sample disruption 3
3.2 DNA extraction 4
3.3 Nanodrop 5

4 DNA fragmentation **5**

5 Gel electrophoresis **6**

6 Reannealing reaction and S1 nuclease digestion **7**

7 Ethanol precipitation of c0t-1 DNA **9**

8 Quantification of c0t-1 DNA **9**

1 Introduction

This is a protocol for preparing c0t-1 DNA from plant material. C0t-1 DNA is used in target capture to reduce non-specific hybridization. This protocol can be completed in 1–2 days. The main steps of the protocol are DNA extraction, fragmentation, gel electrophoresis, S1 nuclease digestion, ethanol precipitation of DNA, and c0t-1 DNA quantification. In the two-day version, samples are stored overnight at -80°C before continuing to ethanol precipitation of DNA and c0t-1 DNA quantification. For optimal compatibility, use plant material from the genotypes used in your target capture experiments. If this is not possible, a good approach is to combine c0t-1 DNA originating from multiple different genotypes.

2 Required Materials

2.1 Equipment

The following equipment is used in this protocol. Some alternative equipment is suggested, however, these are not tested with this protocol and might require some adjustments.

- A set of micropipettes and tips.
- TissueLyser (QIAGEN) or similar machine (mortar and pestle)
- Vortex mixer
- Microcentrifuge capable of at least 10,000 x g
- Hot air oven (optional)
- Sonication device (Bioruptor) or similar machine (enzymatic digestion)
- Nanodrop or similar machine that outputs 260/280 nm and 260/230 nm ratios
- Gel electrophoresis equipment
- Heat blocks (water baths or PCR-machine)
- Refrigerated centrifuge capable of 16,000 x g (cooled to 4°C)

2.2 Reagents and Consumables

E.Z.N.A. SP Plant DNA Kit is used for DNA extractions in this protocol, as it has good performance with conifer samples. Other plant DNA kits should also work.

- DNA extraction kit (E.Z.N.A. SP Plant DNA Kit)
- 2 ml microcentrifuge tubes + stainless-steel beads for TissueLyser
- 0.5 ml Bioruptor Plus microtubes for Bioruptor

- 1.5 ml microcentrifuge tubes
- 0.2 ml PCR tubes
- 3 M NaOH (100 µl per sample) (only if using E.Z.N.A SP Plant DNA kit)
- DNA Gel Loading dye (6X) and 100 bp DNA ladder (1 µl dye per sample, 5–10 µl ladder per gel)
- Agarose
- S1 nuclease and S1 5X Reaction Buffer (less than 1 µl S1 nuclease and 30 µl 5X buffer per sample)
- 5 M NaCL (7 µl per sample)
- EDTA (0.5 M, pH 8.0) (10 µl per sample)
- Sodium acetate (75 µl per sample)
- Glycogen (1 µl per sample)
- 100% ethanol
- Tris-HCL (10 mM, pH 8.0) (10-20 µl per sample)

3 DNA extraction

Sample preparation steps are dependent on the type of tissue and sample. This protocol is optimized for fresh starting material (needles). Some adjustments may be required if frozen or dried plant material is used. Each DNA extraction uses 100 mg of fresh needles and produces around 10,000 ng of c0t-1 DNA. To produce 60,000 ng of c0t-1 DNA for a single exome capture hybridization reaction, 6-7 DNA extractions are required. These sample preparation steps are optimized for conifer needle samples and assume that you are using E.Z.N.A SP Plant DNA kit

3.1 Sample disruption

1. Weigh 100 mg of fresh needles and cut them into 5 mm pieces. Insert pieces into a 2 ml microcentrifuge tube.
2. Add a single large stainless-steel bead into the tube.
3. Add 400 µl SP1 Buffer (E.Z.N.A SP Plant DNA Kit) and 5 µl RNase A (E.Z.N.A SP Plant DNA Kit) into the tube. Vortex at maximum speed to mix thoroughly.
4. Disrupt the samples using Qiagen TissueLyser at maximum speed (30 1/s) for 2 minutes. Vortex the tubes thoroughly and disrupt for another 2 minutes.
5. Incubate the samples at 67 °C in hot air oven, heat block or water bath for 1 hour. Vortex the samples every 15 minutes. The incubation time can safely be extended.

3.2 DNA extraction

The following steps in DNA extraction are copied from E.Z.N.A SP Plant DNA Kit manual revision April 2013 to this protocol for convenience.

1. Remove samples from incubation.
2. Add 140 µl SP2 Buffer. Vortex to mix thoroughly.
3. Let sit on ice for 5 minutes.
4. Centrifuge at maximum speed ($\geq$10,000 x g) for 10 minutes.
5. Insert a Homogenizer Column into a 2 ml Collection Tube.
6. Carefully transfer the supernatant to the Homogenizer Column. Do not disturb or transfer any of the insoluble pellet.
7. Immediately centrifuge at maximum speed for 2 minutes.
8. Transfer cleared lysate to a 1.5 ml microcentrifuge tube. Do not disturb or transfer any of the insoluble pellet. Measure the volume of the lysate.
9. Perform the additional NaOH treatment step by adding 100 µl 3 M NaOH to the HiBind® DNA Mini Column. Centrifuge at 10,000 x g for 30 seconds. Add 100 µl water to the columns and centrifuge at 10,000 x g for 30 seconds. Discard the filtrate.
10. Add 1.5 volumes SP3 Buffer to the 1.5 ml microcentrifuge tube. Vortex immediately to obtain a homogenous mixture. A precipitate may form at this point; it will not interfere with DNA isolation. Passing the mixture through a needle using a syringe or by pipetting up and down 10-15 times may break up the precipitates.
11. Insert a HiBind® DNA Mini Column into a 2 ml Collection Tube
12. Transfer 650 µl sample to the HiBind® DNA Mini Column.
13. Centrifuge at maximum speed for 1 minute.
14. Discard filtrate and reuse the collection tube.
15. Repeat Steps 12-14 until all of the sample has been transferred to the column.
16. Transfer the HiBind® DNA Mini Column to a new 2 ml Collection Tube.
17. Add 650 µl SPW Wash Buffer.
18. Centrifuge at maximum speed for 1 minute
19. Discard filtrate and reuse the collection tube
20. Repeat Steps 17–19 for a second SPW Wash Buffer wash step.
21. Centrifuge the empty HiBind® DNA Mini Column at maximum speed for 2 minutes to dry the column.

22. Transfer the HiBind® DNA Mini Column into a clean 1.5 ml microcentrifuge tube.
23. Add 60 µl Elution Buffer heated to 65°C.
24. Incubate at 65 °C for 5 minutes.
25. Centrifuge at maximum speed for 1 minute.
26. Transfer the eluate from the 1.5 ml microcentrifuge tube back to the HiBind® DNA Mini Column.
27. Repeat Steps 23–24 for a second elution step. (increases yield)

3.3 Nanodrop

Nanodrop is used to quickly assess the DNA concentration and purity. Vortex the samples and measure DNA concentration using Nanodrop (1 µl sample). Use the Elution Buffer from E.Z.N.A SP Plant DNA Kit as a blank in the measurements.

- The 260/280 ratio should be close to 1.8
- The 260/230 ratio shoud not exceed 2.3-2.4.

Do not continue with the protocol, if the ratios differ from the listed values.

4 DNA fragmentation

DNA is fragmented using sonication (Bioruptor UCD-200). The goal is to fragment DNA to 100–500 bp fragments. Sonicator settings and sonication time are optimized for DNA concentration between 700–1000 ng/µl. Higher or lower DNA concentrations may require different settings and sonication times.

Bioruptor tube holder has 12 slots for microtubes, thus 12 samples can be fragmented in a single run. Empty slots in the tube holder must be filled with 0.5 ml Bioruptor Plus microtubes containing 50 uL H_2O. Sonication times in this protocol are optimized for 50–60 µl sample volume.

1. Transfer all of the sample DNA from 1.5 ml microcentrifuge tube to 0.5 ml Bioruptor Plus microtube.
2. Store samples and the 0.5 ml microtubes containing 50 µl H_2O on ice for at least 15 min before sonication.
3. Fill 2/3 of a large beaker with deionized water and add 2 large scoops of ice. Let the water cool down for 10–15 minutes in the beaker. Pour the deionized water into the Bioruptor water bath until water level is almost at the level of the red indicator line.
4. Fill a 250 ml beaker with crushed ice and pour it into the water bath.
5. Adjust water level to the red line by removing water using a small beaker or a pipette.

6. Close the door of the soundproof box containing the water bath. Let the water bath cool for 15 minutes before starting the first sonication round.
7. Vortex the samples for 15 seconds and then centrifuge them for 10 seconds.
8. Insert the samples into the Bioruptor tube holder. Fill empty slots in the tube holder with microtubes containing 50 µl H_2O.
9. Check the amount of ice in the water bath. The layer of ice should be about 5 mm thick. Add more crushed ice if needed.
10. Insert the tube holder into the water bath.
11. Start the fragmentation with the following settings: **Intensity: "M", Multi-timer: 30 seconds on/90 seconds off.**
12. Sonicate the samples for 10 minutes. Follow the fragmentation time using a stop-watch, the total timer in the control unit is not very accurate.
13. Remove the samples from the tube holder. Vortex for 10 seconds. Spin down the samples and the microtubes containing H_2O. Insert them back into the tube holder.
14. Fill entirely a 250 ml beaker with crushed ice and pour it into the water bath.
15. Adjust water level to the red line by removing water using a small beaker or a pipette.
16. Insert the tube holder back into the water bath.
17. Fragment the samples for 6 minutes.
18. Remove the samples from the tube holder. Vortex for 10 seconds and spin down.

5 Gel electrophoresis

Gel electrophoresis is performed to assess the fragment size distribution (Figure 1). Samples should be transferred back to 1.5 ml microcentrifuge tubes after sonication. Tubes can be combined at this step to make the rest of the protocol easier due to having less samples to process. For example, 4 tubes can be combined into 2, 6 tubes into 3, 8 tubes into 4 etc.

1. Measure DNA concentration of the samples using Nanodrop, if tubes were combined.
2. Samples need to be diluted, if DNA concentration is over 1000 ng/µl. Use PCR-grade H_2O for dilutions.
3. Prepare 1% agarose gel.
4. Combine 1 µl of sample, 4 µl of PCR-grade H_2O and 1 µl DNA Gel Loading dye (6X) into a small PCR tube and mix well.

5. Add 5 µl 100 bp DNA ladder into the first (and the last) well on the gel.
6. Add 5 µl of sample DNA - Gel Loading dye mix into a well on the gel.
7. Run the samples on the gel at 100 V for 30–40 minutes.

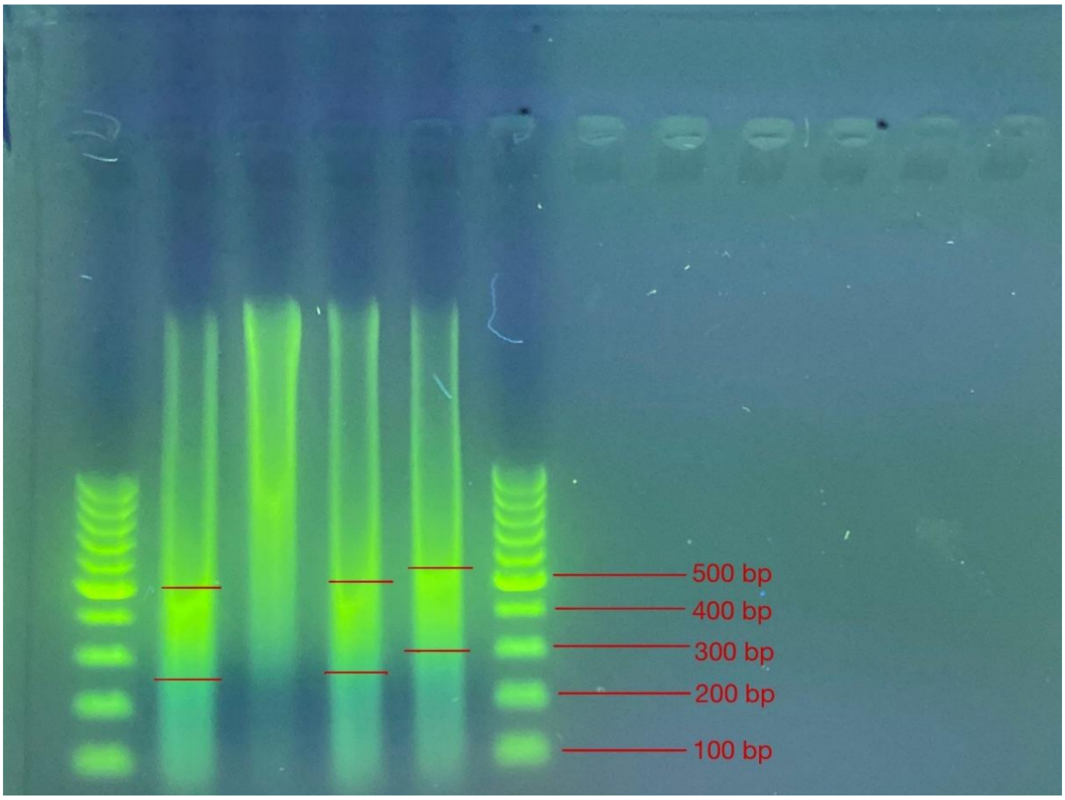

Figure 1: Gel electrophoresis results for successful DNA fragmentation. Most of the fragments are between 250 bp and 500 bp in the 2nd, 4th and 5th well samples. The sample in the 3rd well is too smeared to get proper size estimate. That is due to too high DNA concentration (over 1000 ng/µl). Samples need to be diluted more if smearing occurs and rerun to get a proper size estimate.

6 Reannealing reaction and S1 nuclease digestion

Next step is the reannealing reaction and S1 nuclease digestion. Sample DNA needs to be diluted to 100–600 ng/µl for the next steps of the protocol. DNA is diluted using PCR-grade H_2O and 5 M NaCl to a 0.3 M NaCl contration. NaCl is added to induce breaks in double-stranded DNA. The total volume of diluted DNA with 0.3 M NaCl should be close to 100 µl, as larger volumes require adding higher volumes sodium acetate during a later step, making cleaning c0t-1 DNA more difficult.

Example dilution: 65.5 µl sample DNA (964,69 ng/µl) + 6.6 µl 5M NaCl + 37.9 µl H_2O –> 110 µl 575 ng/µl DNA with 0.3M NaCl concentration

In the following steps sample DNA is first incubated at 95°C to denature it. DNA is allowed to reanneal at 65°C for a short time. Repetitive DNA sequences reanneal faster due to their higher probability of finding complementary strands. The incubation time at 65°C is calculated using the following formula:

T_s = 1 / (diluted sample concentration / 399 gMol) = incubation time in seconds

The remaining single-stranded DNA molecules (non-repetitive DNA) are digested by adding S1 nuclease (Thermo Fisher Scientific) and incubating at +37°C for 15 minutes. The activity of S1 nuclease is then stopped by adding EDTA (0.5 M, pH 8.0) and incubating at +70°C for 10 minutes. Glycogen and sodium acetate are then added to enhance the precipitation of DNA.

The concentration of S1 nuclease stock (Thermo-Fisher) is 100 U/µl. For the S1 nuclease reaction, you need to add 1 U of S1 nuclease per 1 µg of sample DNA. It is recommended to create 1/10 dilution from the stock using PCR-grade H_2O. For example, for 110 µl of 575 ng/µl DNA would require 63.25 U S1 nuclease. Dilute the S1 nuclease before starting the next steps of the procotol and **remember to store the diluted S1 nuclease on ice.** The 5X reaction buffer for S1 nuclease needs to be diluted to 1X for the digestion reaction.

Example S1 nuclease reaction: 110 µl sample DNA (with 0.3 M NaCl) + 6.3 µl 1/10 S1 nuclease dilution + 30 µl 5X reaction buffer + 3.7 µl PCR-grade H_2O = total reaction volume of 150 µl

1. Prepare 95°C, 65°C and 37°C heat blocks, water baths or PCR-machines.
2. Prepare ice water bath, you can use a beaker for this purpose.
3. Incubate diluted sample at 95°C for 10 minutes.
4. Cool the sample by swirling the tube in the ice water bath for 10 seconds.
5. Immediately incubate the sample at 65°C for the calculated incubation time (T_s).
6. Remove the sample and put in on ice. Set the 65°C heat block to 70°C.
7. Add the calculated amount of H_2O, 5X Buffer and 1/10 S1 nuclease dilution. Vortex to mix the reagents.
8. Incubate at 37°C for 15 minutes.
9. Stop the activity of S1 nuclease by adding 2 µl EDTA (0.5 M, pH 8.0) per every 30 µl of sample.
10. Immediately incubate at 70°C for 10 minutes.
11. Remove the sample from incubation and add 1 µl glycogen.
12. Add 0.5 volumes of sodium acetate.
13. Add 2.5 volumes of 100% ethanol.
14. Store the sample at -80°C for at least few hours, preferably for overnight.

7 Ethanol precipitation of c0t-1 DNA

In this step of the protocol c0t-1 DNA is purified using ethanol precipitation.

1. Prepare 70% ethanol (450 µl per sample) and store at -20°C.
2. Pre-cool refrigerated centrifuge capable of 16,000 x g to 4°C.
3. Centrifuge the sample at 4°C 16,000 x g for 30 minutes.
4. Remove the supernatant. Do not disturb or transfer any of the insoluble pellet.
5. Add 150 µl cold 70% ethanol. Vortex briefly.
6. Centrifuge the sample at 4°C 16,000 x g for 2 minutes.
7. Remove the ethanol. Do not disturb or transfer any of the insoluble pellet.
8. Repeat Steps 5–7 for a second and a third ethanol wash.
9. Spin briefly to collect any traces of ethanol to the bottom of the tube.
10. Remove the last traces of ethanol by pipetting.
11. Open the cap of sample tube and turn the tube upside down onto a paper towel. Let the DNA pellet dry for 15 minutes.
12. Add 10–20 µl Tris-HCl (10 mM, pH 8.0) and dissolve the DNA pellet by pipetting up and down or by vortexing. Adding 10 µl Triss-HCl is recommened if many samples are going to be multiplexed in the exome capture, to reduce the total reaction volume.

8 Quantification of c0t-1 DNA

1. Vortex c0t-1 DNA samples and measure DNA concentration using Nanodrop (0.5 µl sample). Use Triss-HCl as a blank in the measurements.
2. Plot the results with the Nanodrop software. You should see only one large peak at wavelength of 260 nm. Possible secondary peak at 230 nm is caused by salt contamination. See Figure 2 below for reference. Repeat ethanol wash steps, if clear salt contamination is visible.
3. Calculate the mass of C0t-1 DNA in the tube based on the concentration from Nanodrop measurement and the volume of Triss-HCl used. Remember that 0.5 µl C0t-1 DNA was used in the Nanodrop measurement.
4. Combine c0t-1 DNA samples to get the desired amount (60,000 ng) of c0t-1 DNA into a single tube.
5. Store the c0t-1 DNA tubes at -20°C before usage.

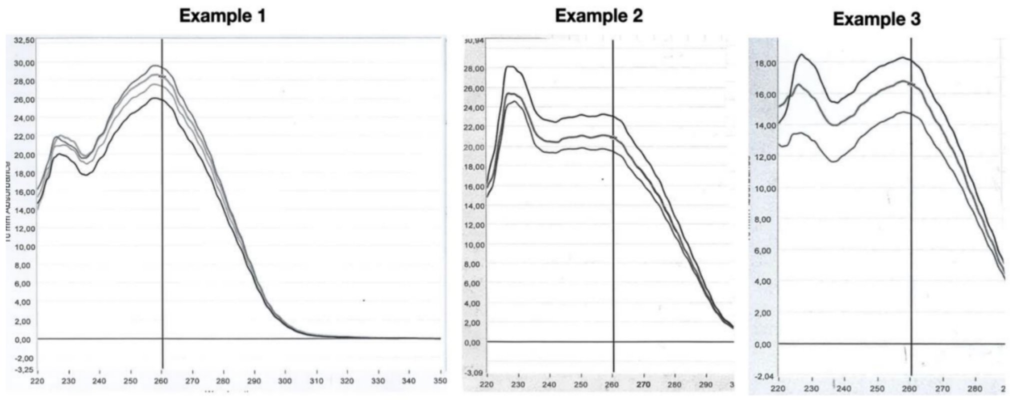

Figure 2: Nanodrop plots used in quantification of C0t-1 DNA. Example 1 represents successful C0t-1 DNA, having the main peak at wavelength of 260 nm. The secondary peak at wavelength of 230 nm represents a small amount of salt contamination, which is still acceptable. Additional cleaning steps would cause dramatic loss in the amount of C0t-1 DNA and only a minor decrease in the salt contamination. Example 2 represents C0t-1 DNA with large amount of salt contamination. The peak at 230 nm is much higher than the peak at 260 nm. Additional cleaning rounds are required. Example 3 also represent C0t-1 DNA with salt contamination. Contamination is not as high as in example 2. Additional cleaning rounds are still required. Peak at 230 nm in the examples 2 and 3 are most likely due to salts, but can also be caused by traces of glycogen or ethanol. Allowing the DNA pellet to dry for a longer time can help with trace ethanol.

References

This protocol is based on the following publications:

[1] Evan McCartney-Melstad, Genevieve G Mount, and H Bradley Shaffer. “Exon capture optimization in amphibians with large genomes”. In: *Molecular ecology resources* 16.5 (2016), pp. 1084–1094.

[2] Michael S Zwick et al. “A rapid procedure for the isolation of C 0 t-1 DNA from plants”. In: *Genome* 40.1 (1997), pp. 138–142.
