# Supplementary material for: Optimising Exome Captures in Species With Large Genomes Using Species‐Specific Repetitive DNA Blocker
Source: Mol Ecol Resour. 2024 Dec 18;25(3):e14053. doi: 10.1111/1755-0998.14053 (PMC11887611; doi:10.1111/1755-0998.14053)
Supplement: Supplementary file 5 — FIGURE S1. Sequencing coverage for target regions plotted as a kernel density estimate (KDE) plot. [file MEN-25-e14053-s004.pdf]

Supplemental figures

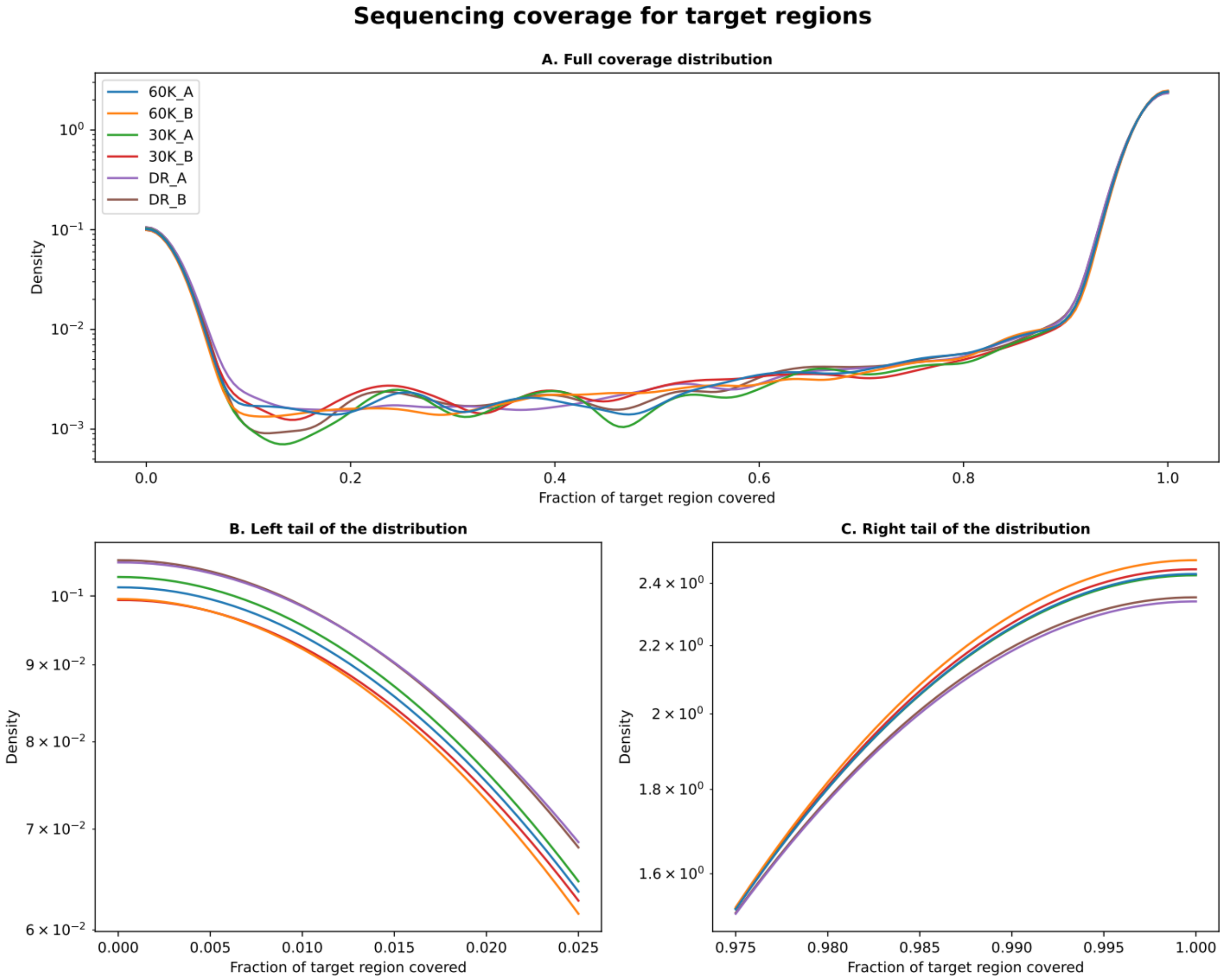

Supplemental figure 1: Sequencing coverage for target regions plotted as a kernel density estimate (KDE) plot. A) Full coverage distribution, B) Zoomed view on the left tail of the distribution, and C) Zoomed view on the right tail of the distribution. Y-axis is log scaled.
